# Regional lymph node evaluation in pediatric conventional melanoma subtype: a single-center 10-year review

**DOI:** 10.1007/s00383-024-05646-8

**Published:** 2024-03-05

**Authors:** Pattamon Sutthatarn, Andrew M. Davidoff, Armita Bahrami, Celine Richard, Bhatia Shalini, Teresa C. Santiago, Barry L. Shulkin, Alberto S. Pappo, Abdelhafeez Abdelhafeez

**Affiliations:** 1 Department of Surgery, St. Jude Children’s Research Hospital, Memphis, TN 38105 USA; 2 Department of Surgery, Faculty of Medicine, Chulalongkorn University and King Chulalongkorn Memorial Hospital, Bangkok, Thailand; 3 Division of Pediatric Surgery, Department of Surgery, University of Tennessee Health Science Center, Memphis, TN 38105 USA; 4 Department of Pathology and Laboratory Medicine, Emory University School of Medicine, Atlanta, GA 30322 USA; 5 Department of Otolaryngology, University of Tennessee Health Science Center College of Medicine, Memphis, USA; 6 Division of Otolaryngology, St. Jude Children’s Research Hospital, Memphis, TN 38105 USA; 7 Department of Biostatistics, St. Jude Children’s Research Hospital, Memphis, TN 38105 USA; 8 Department of Pathology, St. Jude Children’s Research Hospital, Memphis, TN 38105 USA; 9 Department of Diagnostic Imaging, St. Jude Children’s Research Hospital, Memphis, TN 38105 United States; 10 Department of Oncology, St. Jude Children’s Research Hospital, Memphis, TN 38105 United States

**Keywords:** Melanoma, Sentinel lymph mode biopsy, Spitz melanoma, Pediatric melanoma

## Abstract

**Purpose:**

To assess the prognostic and therapeutic significance of sentinel lymph node biopsy (SLNB) and completion lymph node dissection (CLND) in pediatric conventional melanoma (CM), while evaluating potential predictive factors for outcomes.

**Methods:**

We conducted a retrospective analysis of medical records spanning 2009–2020, focusing on patients aged 18 or younger with localized cutaneous conventional melanoma.

**Results:**

Among the 33 patients, SLNB detected metastasis in 57.6% of cases, with 52.6% undergoing CLND. Positive SLN patients had higher relapse risk (HR 5.92; 95% CI 1.27–27.7; *P* = 0.024) but similar overall survival (HR 3.19; 95% CI 0.31–33.1, *P* = 0.33).

No significant differences in disease-free survival (DFS) and OS were found between patients who underwent CLND and those who did not (HR 1.91; 95% CI 0.49–7.43, *P* = 0.35, and HR 0.52; 95% CI 0.03–8.32, *P* = 0.64, respectively). Univariate analysis showed age at diagnosis (*P* = 0.02) correlated with higher* recurrence risk, with a 21% hazard increase per additional year of age.*

**Conclusions:**

Positive SLN status and age at diagnosis were associated with worse DFS in CM patients. Our study did not find any prognostic or therapeutic value in CLND for pediatric melanoma. Further multicenter trials are needed to confirm our single-institution experience.

**Level of evidence:**

Level IV.

## Introduction

Although melanoma is a rare entity in childhood and adolescence, accounting for less than 1% of all pediatric malignancies [1–4], it is the deadliest pediatric skin malignancy [5]. Furthermore, it is the most common pediatric skin cancer, with an incidence that increases with age, rising to 4% of all malignancies in adolescents age 15–19 years [6, 7]. Three types of melanomas develop in the pediatric cohort: conventional melanoma (CM), melanoma that arises in a large/giant congenital melanocytic nevus, and Spitz melanoma (SM), the latter being the most common pediatric type [8, 9]. CM, also known as “adult-type melanoma,” shares the same causes and risk factors as in adults, and, therefore, treatment options are often based on adult protocols [8]. In contrast, SM exhibits different spreading patterns, and identification of prognostic factors remains challenging [8, 10, 11].

Like adult melanomas, most pediatric melanomas are localized, and their prognosis varies with age and stage of disease [12–14]. The more advanced the stage, the poorer the outcome [15, 16]. However, the prognostic significance of histologic risk factors identified for adult cases (e.g., vascular invasion, mitotic activity) has not been directly examined in pediatric tumors [17, 18]. More specifically, while traditional prognostic parameters for adult cutaneous melanoma, i.e., depth of the tumor (or Breslow thickness) and nodal metastases [19, 20] carry different predictive values in pediatric SM lesions, they have not been specifically evaluated in pediatric CM lesions [18, 21]. Efforts to identify reliable prognostic factors for pediatric melanoma were recently directed toward the evaluation of candidate immunohistochemical and molecular markers. This revealed the TERT (telomerase reverse transcriptase) oncogene promoter mutation in pediatric SM to be associated with a poorer prognosis [17, 21].

Uncertainty remains regarding the optimal management of regional lymph nodes in pediatric melanoma [2, 22]. Although sentinel lymph node (SLN) involvement has been validated as a prognostic marker in adult patients with melanoma [19, 23], its impact on overall survival (OS) in the pediatric population is unclear [9, 24, 25]. Due to its rarity in childhood and adolescence, pediatric melanoma is underrepresented in clinical trials, and pediatric oncologic teams have aligned their care strategies to guidelines developed in adults. Most melanoma studies in pediatric population had both CM and SM which are two entities that behave differently. In this study we aim to focus on patients with CM only.

**Fig. 1:**
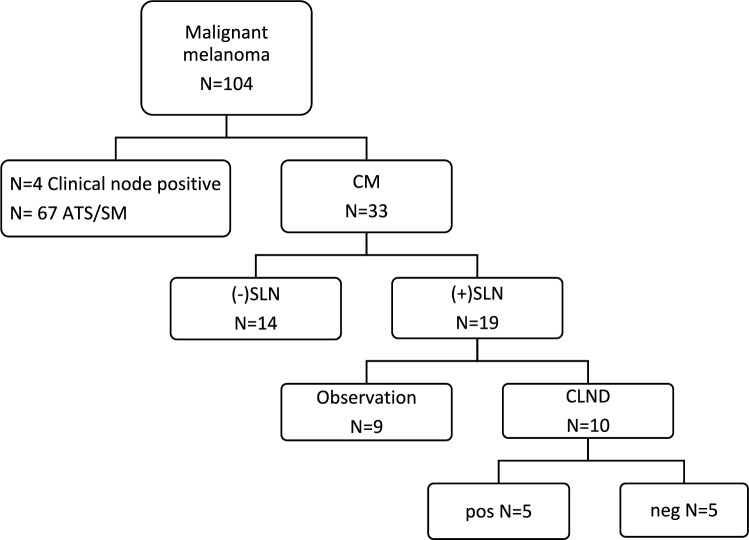
Flow Diagram of children diagnosed with cutaneous melanoma treated at our hospital. *ATS/SM* atypical spitzoid melanoma/spitzoid melanoma, *CM* conventional melanoma, *SD* standard deviation, *mm* millimeter, *SLN* sentinel lymph node, *(+)* positive, *(−)* negative

The aim of this study was two-fold. The primary objective was to provide a qualitative analysis of the prognostic role and impact of sentinel lymph node biopsy (SLNB) and completion lymph node dissection (CLND) on the outcome of pediatric conventional melanoma. Our secondary objective was to examine the different prognostic factors associated with survival and recurrence of disease (Fig. 1).

## Methods

### Study design and data source

This retrospective single-center cohort study and related protocol were approved by the Institutional Review Board at St. Jude Children’s Research Hospital. We included pediatric patients aged 18 years or younger with a diagnosis of localized cutaneous melanoma of the conventional subtype without clinically apparent regional node involvement, seen at our institution between 2009 and 2020. We reanalyzed all pathologic data and slides for patients referred to our center who had their primary surgery and/or SLNB at another institution. We excluded patients with non-CM subtype, clinically detectable regional nodal and/or distant metastases. Demographic data (i.e., age at the time of diagnosis, sex, race), primary tumor characteristics (tumor location, type of melanoma, Breslow thickness, presence of ulceration, mitotic index, resection margin), nodal status, regional lymph node management, recurrence rate, and survival were collected from patients’ medical records. Diseases were staged according to the American Joint Committee on Cancer (AJCC) staging system [20].

### Statistical analysis

First, the pediatric patients who underwent SLNB were categorized based on the results of the procedure, specifically the SLN status. Then, the subgroup of patients identified with a positive SLN were classified based on whether they underwent nodal observation or CLND. Descriptive statistics included evaluating central tendency using mean ± standard deviation (SD) and median with interquartile ranges (IQR). Continuous variables were compared using the two-tailed Student’s *t* test or Wilcoxon rank sum test, while categorical variables were compared using the chi-square or Fisher exact test.

The primary endpoints of our analysis were OS, which was defined by time from diagnosis of CM to death from any causes, and disease-free survival (DFS) defined by time from complete resection to recurrence. OS and DFS were analyzed using Kaplan–Meier curves, and they were compared using a log-rank test. Prognostic factors for both OS and DFS were examined utilizing the log-rank test and the Cox Proportional Hazards (Cox-PH) model in univariate analysis. Results are reported as a *P* value or hazard ratio (HR) with 95% confidence interval (CI). Statistical analysis was performed using (R version 3.6.2). A *P* value < 0.05 was considered significant.

## Results

### Study population and tumor characteristics

Among the 100 patients seen at our institution who underwent SLNB for a melanocytic lesion, 67 had ATS/SM subtype and were thus excluded from the study. The final cohort included 33 patients with conventional melanoma. Among the 19 (57.6%) patients with positive SLN, 10 (52.6%) underwent CLND.

There were 14 (42%) female patients, with a median age at diagnosis of 13 years (IQR = 2–21). All the patients were white. Fifteen (45%) patients had primary lesions on the truncal area. Nodular subtype was the most common (*n* = 8) histologic subtype. The mean tumor depth was 3.47 mm (SD = 2.54); 19 (57%) of the patients had a tumor depth exceeding 2 mm. The primary tumor of 30 (91%) patients had a mitotic rate of at least 1/mm^2^ (Table 1).

**Table 1:** Patient and tumor characteristics of patients with pediatric CM cutaneous melanoma

Characteristic	No. (%)^a^
Age at diagnosis (years)	
Mean (SD)	12.55 (4.02)
Median (range)	13 (2, 21)
Sex	
Female	14 (42%)
Male	19 (58%)
Race	
White	33 (100%)
Histology subtypes	
Acral lentiginous	1 (3%)
Folliculocentric	1 (3%)
Nevoid	4 (12%)
Nodular	8 (24%)
Pigment synthesizing	1 (3%)
Superficial spreading	5 (15%)
Unknown	13 (40%)
Primary tumor location	
Extremity	9 (27%)
Head or neck	9 (27%)
Trunk	15 (45%)
Tumor depth	
T1	3 (9%)
T2 (1.01–2.00 mm)	11 (33%)
T3 (2.01–4.00 mm)	9 (27%)
T4 (> 4.00 mm)	10 (30%)
Depth (mm)	
Mean (SD)	3.47 (2.54)
Median (range)	2.36 (0.42, 9.4)
Ulceration	
Yes	14 (42%)
No	19 (58%)
Mitotic counts	
Mean	6.4 (6.49)
Median	4 (0,26)
Mitosis	
Absence	3 (9%)
Presence	30 (91%)

*CM* conventional melanoma, *mm* millimeter, *N/A* not available, *SD* standard deviation

^a^Values indicate the number of patients (%), unless otherwise indicated

### Sentinel lymph node evaluation

Of the 33 patients, 19 had lymph node metastasis (Table 2). The median follow-up was 5.16 years (95% IQR 2.92–9.16) in the negative SLN group and 4.74 years (95% IQR 2.07–5.77) in the positive SLN group. For all patients with extremity lesions (nine patients), the SLN, as localized with a nuclear medicine scan, was consistently in the ipsilateral groin for lower extremities and in the ipsilateral axilla for the upper extremities. None of the patients with a T1 lesion or a tumor depth of less than 1 mm had a positive SLN.

**Table 2:** Patients and tumor characteristics of those with positive vs negative sentinel lymph nodes

Characteristic	Positive SLN (*n* = 19) *n* (%)^a^	Negative SLN (*n* = 14) *n* (%)^a^	*P* value
Age at diagnosis			0.21
Mean (SD)	13.16 (3.95)	11.71 (4.1)	
Median (range)	14 (2, 21)	11.5 (4, 18)	
Age at diagnosis			> 0.99
< 10 years	3 (16%)	3 (21%)	
≥ 10 years	16 (84%)	11 (79%)	
Sex			0.45
Female	7 (37%)	7 (50%)	
Male	12 (63%)	7 (50%)	
Primary tumor location			0.062
Extremity	3 (16%)	6 (43%)	
Head or neck	4 (21%)	5 (36%)	
Trunk	12 (63%)	3 (21%)	
Depth (mm)			0.25
Mean (SD)	3.75 (2.43)	3.1 (2.72)	
Median (range)	3.3 (1.3, 9.4)	2.02 (0.42–9.20)	
Tumor depth			0.28
T1	–	3 (21%)	
T2 (1.01–2.00 mm)	7 (37%)	4 (29%)	
T3 (2.01–4.00 mm)	6 (32%)	3 (21%)	
T4 (> 4.00 mm)	6 (32%)	4 (29%)	
N/A			
Ulceration			0.17
Yes	10 (53%)	4 (29%)	
No	9 (47%)	10 (71%)	
Mitosis			0.56
Absence	1 (5%)	2 (14%)	
Presence	18 (95%)	12 (86%)	
Mitotic counts			0.37
Mean	6.79 (6.38)	5.87 (6.84)	
Median	5 (0, 26)	3 (0, 21)	
Margin status^b^			0.016
1–5 mm	7 (37%)	2 (14%)	
5–10 mm	2 (11%)	4 (29%)	
> 10 mm	4 (21%)	8 (57%)	
Free	6 (32%)	–	
Adjuvant treatment^c^			< 0.001
Yes	17 (89%)	2 (14%)	
No	2 (11%)	12 (86%)	
Median number (range) of SLN identified per patient	3 (1,7)	2 (1,13)	0.37

*CM* conventional melanoma, *SD* standard deviation, *mm* millimeter, *SLN* sentinel lymph node

^a^Values indicate the number of patients (%), unless otherwise indicated

^b^Margin status indicates the size of the resection margin

^c^Adjuvant treatment included interferon alpha or immunotherapy

The hazard of relapse in the positive SLN group was 5.92 times higher than the risk in the negative SLN group (HR 5.92; 95% CI 1.27–27.7; *P* = 0.02) (Table 3).

**Table 3:** Univariate hazard ratios for melanoma recurrence and death of overall 33 patients

Prognostic factors	Recurrence factor		Survival factor	
	HR (95% CI)	*P* value	HR (95% CI)	*P* value
SLN (pos vs neg)	5.92 (1.27–27.7)	0.024	3.19 (0.31–33.1)	0.33
Age at diagnosis				
(≥ 10 years vs < 10 years)	3 (0.38–23.4)	0.29	–^a^	
Age (range)	1.21 (1.02–4.44)	0.028	–^a^	
Primary tumor location				
Trunk vs extremity	7.74 (0.9–66.7)	0.062	–^a^	
Head or neck vs extremity	6.98 (0.69–70.4)	0.1	–^a^	
Sex				
(Male vs female)	0.87 (0.29–2.62)	0.81	0.82 (0.11–5.93)	0.84
Ulceration				
(Yes vs no)	1.93 (0.64–5.77)	0.24	0.37 (0.04–3.64)	0.36
Median tumor depth (range)	1.15 (0.96–1.4)	0.14	0.79 (0.48–1.3)	0.36
Margin status^b^ (vs 10 mm)				
< 5 mm	1.31(0.33–5.25)	0.71	–^a^	
≥ 5 mm to < 10 mm	0.87(0.16–4.81)	0.88	–^a^	
Mitotic counts (presence vs absence)	1.6 (0.21–12.4)	0.65	–^a^	

*CI* confidence interval, *CLND* complete lymph node dissection, *CM* conventional melanoma, *HR* hazard ratio, *neg* negative, *pos* positive, *Q* quartile, *SD* standard deviation, *SLN* sentinel lymph node

^a^The samples were too small to analyze HR

^b^Margin status indicates the size of the resection margin

**Fig. 2:**
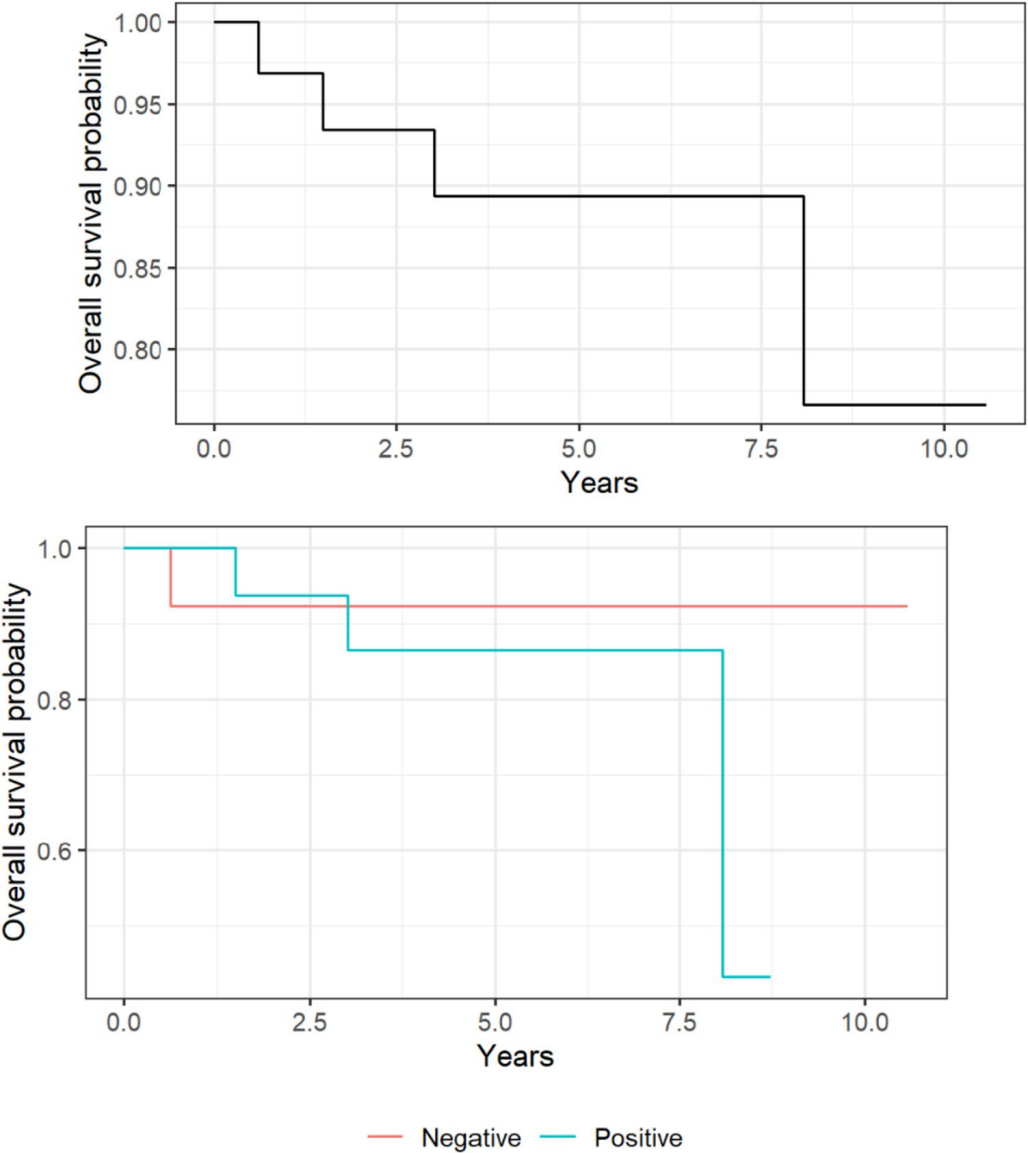
The Kaplan–Meier survival curves illustrate the overall survival probability for the entire cohort (upper panel) and as a function of sentinel lymph node status (lower panel)

**Fig. 3:**
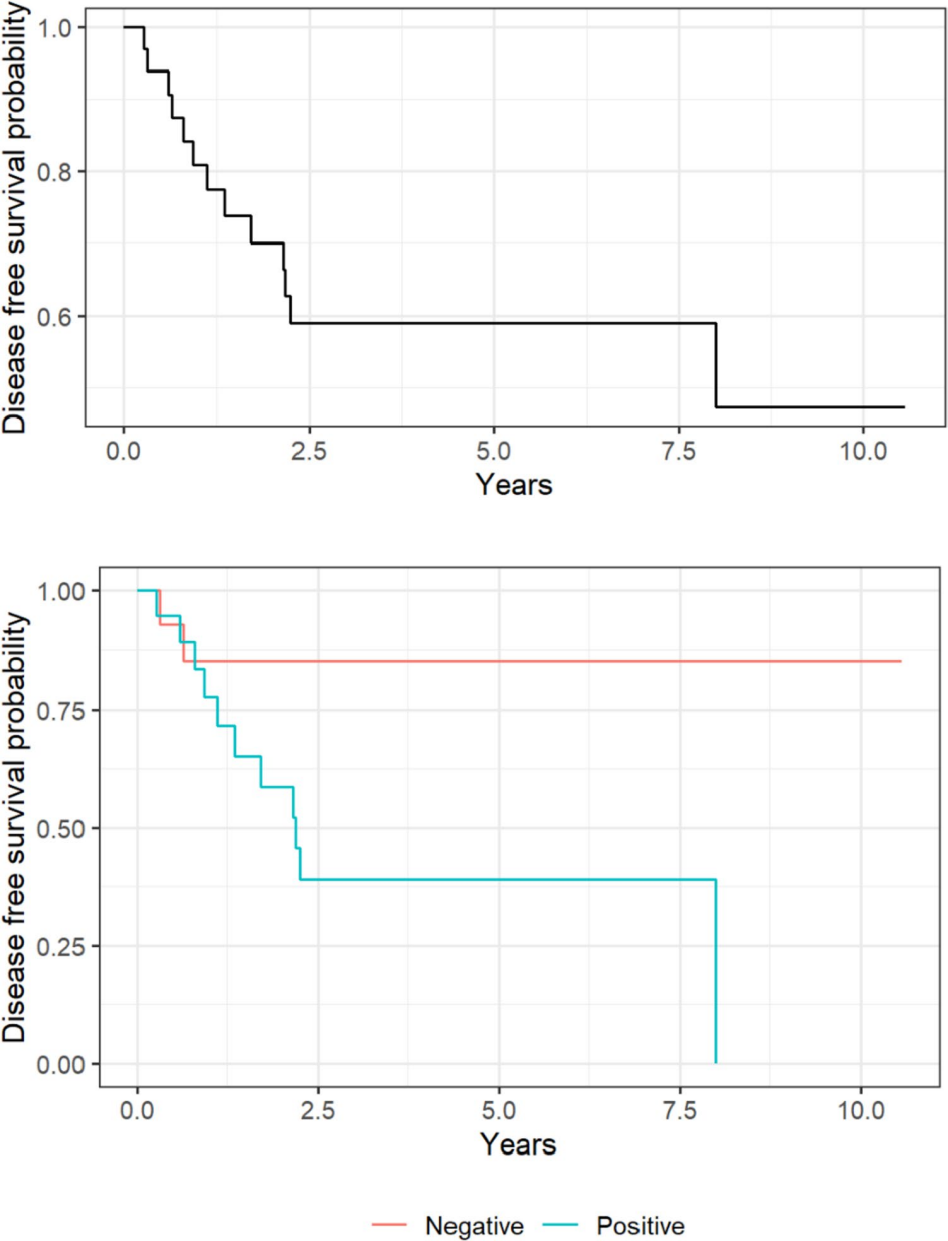
Disease-free survival probability for the entire cohort (upper panel) and as a function of sentinel lymph node status (lower panel)

There was no difference in OS between the patients with negative SLN and those with positive SLN (HR 3.19; 95% CI 0.31–33.1, *P* = 0.33), with a 5-year OS rate of 92.3% (95% CI 78.9–100%), and 86% (95% CI 70.7–100%), respectively; Fig. 2. Of note, one patient had anaphylaxis secondary to methylene blue. This patient completely recovered after management with adrenaline and steroids. DFS changed significantly with age at diagnosis HR (95% CI) = 1.2 (1.02–1.44), *P* = 0.03, and with a 5-year DFS of 85.1% (95% CI: 68–100%) and of 39.1% (95% CI: 21.1–72.3%) in the negative SLN and positive subgroup, respectively; Fig. 3.

### Complete lymph node dissection vs observation for sentinel-node metastasis

Table 4 shows the tumor characteristics and demographics of patients who underwent CLND vs observation for a positive SLN status. Of the 19 patients who had a positive SLN, 10 individuals (53%) underwent complete CLND, while the remaining 9 patients (47%) were managed through observation-alone. The decision to perform a CLND was influenced by the presence of high-risk clinical features. Notably, patients who underwent CLND were slightly older (*P* = 0.043) and had more ulceration (*P* = 0.023) compared to those who received conservative treatment (Table 5). As a result of CLND, 5 (50%) patients were found to have further nodal metastasis. Median follow-up was 5.45 years (IQR 4.87–6) in the CLND group and 2.6 years (IQR 1.42–3.66) in the observation group.

**Table 4:** Patient and tumor characteristics of those who underwent CLND vs no CLND

Characteristic	CLND (*n* = 10) *n* (%)^a^	Non CLND (*n* = 9) *n* (%)^a^	*P* value
Age at diagnosis (years)			0.043
Mean (SD)	15 (2.83)	11.11 (4.14)	
Median (range)	14 (11, 21)	13 (2, 15)	
Sex			> 0.99
Female	4 (40%)	3 (33%)	
Male	6 (60%)	6 (67%)	
Primary tumor location			0.18
Extremity	–	3 (33%)	
Head or neck	3 (30%)	1 (11%)	
Trunk	7 (70%)	5 (56%)	
Depth (mm)			0.97
Mean (SD)	4.08 (3.04)	3.38 (1.63)	
Median (range)	2.63 (1.3, 9.4)	3.5 (1.55, 7)	
Tumor depth			0.62
T1	–	–	
T2 (1.01–2.00 mm)	4 (40%)	3 (33%)	
T3 (2.01–4.00 mm)	2 (20%)	4 (44%)	
T4 (> 4.00 mm)	4 (40%)	2 (22%)	
Ulceration			0.023
Yes	8 (80%)	2 (22%)	
No	2 (20%)	7 (78%)	
Mitosis			0.47
Absence	–	1 (11%)	
Presence	10 (100%)	8 (89%)	
Mitotic counts			0.15
Mean	8.7 (7.56)	4.67 (4.21)	
Median	6.5 (2, 26)	3 (0, 13)	
Margin status^b^			0.78
1–5 mm	4 (40%)	3 (33%)	
5–10 mm	1 (10%)	1 (11%)	
> 10 mm	3 (30%)	1 (11%)	
Free	2 (20%)	4 (44%)	
Adjuvant treatment^c^			0.21
Yes	10 (100%)	7 (78%)	
No	–	2 (22%)	
Median number (range) of SLN identified per patient	3 (1, 7)	3 (1, 4)	0.79

*CM* conventional melanoma, *SD* standard deviation, *mm* millimeter, *SLN* sentinel lymph node

^a^Values indicate the number of patients (%), unless otherwise indicated

^b^Margin status indicates the size of the resection margin

^c^Adjuvant treatment included interferon alpha or immunotherapy

**Table 5:** Univariate hazard ratios for melanoma recurrence and death of positive SLN patients (*N* = 19)

Prognostic factors	Recurrence factor		Survival factor	
	HR (95% CI)	*P* value	HR (95% CI)	*P* value
CLND (yes vs no)	1.98 (0.28–14.2)	0.35	0.52 (0.03–8.32)	0.64
Age at diagnosis				
(≥ 10 years vs < 10 years)	0.76 (0.09–6.5)	0.8	–^a^	
Primary tumor location				
Trunk vs extremity	0.51 (0.06–4.63)	0.55	–^a^	
Head or neck vs extremity	0.9 (0.09–9.5)	0.93	–^a^	
Sex				
(Male vs female)	1.88 (0.48–7.38)	0.37	1.45 (0.13–16.3)	0.76
Ulceration				
(Yes vs no)	1.63 (0.46–5.81)	0.45	0.47 (0.04–5.24)	0.54
Median tumor depth (range)	1.12 (0.86–1.47)	0.38	–^a^	
Margin status^c^ (vs 10 mm)				
< 5 mm	0.37 (0.03–4.13)	0.42	–^a^	
≥ 5 mm to < 10 mm	–	–		
Mitotic counts				
(presence vs absence)	0.37 (0.04–3.17)	0.36	–^a^	

^a^The samples were too small to analyze HR

^b^Margin status indicates the size of the resection margin

*CI* confidence interval, *CLND* complete lymph node dissection, *CM* conventional melanoma, *HR* hazard ratio, *neg* negative, *pos* positive, *Q* quartile, *SD* standard deviation, *SLN* sentinel lymph node

There was no difference in DFS and OS between the patients who underwent CLND and those who did not undergo CLND. The hazard ratios for DFS and OS were (HR 1.91 (0.49–7.43), *P* = 0.35) and (HR 0.52; 95% CI 0.03–8.32, *P* = 0.64), respectively. Two (20%) patients had complications related to the CLND that included a seroma and a wound infection (n = 1 each).

### Adjuvant therapy

Adjuvant therapy was administered to 19 patients. Within this group, 17 patients had positive SLN, while 2 individuals presented with negative SLN. In the positive SLN subgroup, 8 patients opted for immunotherapy and 11 underwent treatment with interferon. Fatalities were observed for three patients who received interferon. In the negative SLN subgroup, two patients received interferon.

Among the 14 individuals who did not undergo adjuvant treatment, 2 had positive SLN, resulting in 1 case of recurrence at stage T3aN0. The remaining 12 patients in this subset exhibited negative SLN, and 1 patient in this category succumbed to stage T4N0M1.

### Predictor analyses

Four patients died of melanoma. All these patients experienced a recurrence of their tumor and 3 had a positive SLNB (Table 6). Univariate analysis was performed to identify prognostic factors associated with mortality and recurrence (Table 3). The recurrence of trunk or head and neck lesions tended to be greater than that of lesions on the extremities (HR = 7.74; 95% CI 0.9–66.7; *P* = 0.062) and HR 6.98 (95% CI 0.69–70.4; *P* = 0.1) for the truncal lesion and head/neck lesion, respectively. None of the factors were correlated with survival overall.

**Table 6:** Characteristics of the four patients with pediatric melanoma who died

#	Characteristic									
	Sex, age at Dx (years)	Histology	Primary tumor location	Tumor stage	SLNB (status)	CLND (result)	Margin (mm)	Mitototic count^a^	Genomics	Time to death^b^ (months)
1	M, 13	Nodular	Truncal	T2a	Pos (1/2), subcapsular	N	Free	3	n/a	37
2	F, 16	Melanoma	Truncal	T3a	Pos (2/5), subcapsular	Y (neg)	0.5	3	n/a	96
3	M, 14	Superficial spreading	Truncal	T2b	Pos (1/1), extracapsular	Y (neg)	7	5	BRAF	17
4	F, 13	Folliculocentric	Head/neck	T4a	neg, 0/5	N	8	> 20	BRAF and P16 deletion	6

^a^Mitotic counts indicate number of mitosis per mm^2^

^b^Time to death indicates the period from diagnosis to mortality

*CLND* complete lymph node dissection, *Dx* diagnosis, *F* female, *M* male, *pos* positive, *N* no, *neg* negative, *Pt No.* patient number, *SLNB* sentinel lymph node biopsy

## Discussion

This study revealed that CM pediatric patients with a positive SLN were significantly more likely to experience melanoma recurrence than those with a negative SLN. It also appears that, for patients with positive SLNB, CLND did not change the outcome; however, the small sample size and the limited statistical power are acknowledged.

These results were consistent with the MSLT (Multicenter Selective Lymphadenectomy Trial-I) and MSLT-II studies in the adult population [23, 26]. According to the MSLT-I, there was no significant treatment-related difference in 10-year melanoma-specific survival (MSS) among the overall patient population. However, DFS and MSS were significantly improved in the SLNB group with intermediate lesions, which were defined as 1.20- to 3.50-mm depth. In our study, none of the patients with lesions less than 1.01 mm had a positive SLN.

This study also revealed that SLNB status is a prognostic indicator for melanoma recurrence and death of patients with lesions greater than or equal to 1.2-mm depth. Nevertheless, CLND did not increase MSS among patients with melanoma and SLN metastases [6, 15]. A previous report on pediatric melanomas from Kim et al. (2016) revealed that SLNB does not confer survival benefit to children with melanoma [2]. A study of the Surveillance, Epidemiology, and End Results database (SEER) showed that a positive SLN was associated with a poorer melanoma-specific free survival (89% vs 100% at 84 months, *P* = 0.04) [2]. However, it is unclear if a CLND should be performed in all SLN-positive patients, especially when considering the high risk of lymphedema [26] and other associated postoperative complications. Conservative clinical management of positive lymph nodes in pediatric melanoma is supported by the MSLT-II study, which found that observation of SLN-positive patients is safe in the low-risk group, which includes patients who do not have any extracapsular spread/extension, any concomitant microsatellites of the primary tumor, more than three involved nodes, or any involvement of more than two nodal basins in patients with immunosuppression [19, 26].

Currently, the mainstay of treatment of pediatric melanoma is to perform a wide local excision, potentially including a SLN [22]. The controversy is whether these patients need to undergo SLNB or CLND, per the adult guidelines [2, 3, 22]. SLNB is not recommended for patients who have lesions less than 0.8 mm, without ulceration, and categorized as T1a, because the incidence of positive SLN is less than 5% [23]. Pediatric SM has a very different behavior and a better prognosis than CM or melanoma in the adult population [11, 17, 18, 21]. Thus, the staging criteria being used in adults with SM, such as Breslow thickness or nodal metastasis, do not have the same prognostic value in pediatric patients.

Although SLNB does not improve MSS in the pediatric population, it can be used as a prognostic indicator of poorer outcomes [5]. This is strengthened by a study of 310 pediatric patients (< 20 years old) with melanoma whose lesion had a Breslow depth exceeding 0.75 mm that revealed an impact of SLNB positivity on MSS (100% for the group with negative SLNB vs 89% for the group with positive SLNB) [2].

Based on previous publications and our findings, it is still recommended to perform a SLNB in lesions that are 0.8 mm deep with ulceration or deeper than 0.8 mm with or without ulceration [19]. However, we do not recommend CLND in low-risk CM without risk factors associated with extranodal metastasis (i.e., age ≥ 10 years, presence of ulceration, mitotic activity > 5/mm^2^ [21]) or *TERT* promotor mutation [11, 17]. Our institutional protocol for patients with low-risk disease but a positive SLN includes clinical examination every 4 months during the first 2 years, every 6 months during years 3 through 5, and annually thereafter. Ultrasonographic assessment of the sentinel-node basin is performed at each visit for the first 5 years. Abnormal ultrasonographic findings include a lymph node length-to-depth ratio of less than 2 cm, a hypoechoic center, an absence of hilar vessels, or focal nodularity with increased vascularity [26].

We found, on our univariate analysis, that a positive SLNB and age at diagnosis, as a continuous data, were associated with higher recurrence in patients with CM. The limited sample of patients prevented multivariable analysis. According to the previous studies the prognosis of childhood melanoma is associated with lesion thickness, stage of the disease, truncal location, and presence of ulceration [2, 5, 22, 27]. Controversy persists in terms of how wide the resection should be in the pediatric population. According to the adult recommendations, the margin is determined by the depth of the lesion. However, we do not usually perform local excision wider than 1 cm due to the limitation of body surface area in children. We found that recurrence and mortality rates were higher in patients whose excision margins were < 5 mm, however, the difference was not statistically significant.

## Conclusion

Positive SLN status and age at diagnosis were associated with worse DFS in CM patients. Our study did not find any prognostic or therapeutic value in CLND for pediatric melanoma. However, further multicenter trials are needed to confirm our single-institution experience. In extremity cutaneous melanoma, the location of the SLN is always predictable, being the axilla for the upper-extremity melanoma and the groin for the lower-extremity melanoma.

## Data Availability

The data supporting this study's findings are not openly available due to reasons of sensitivity and are available from the corresponding author upon reasonable request. Data are located in controlled access data storage at St Jude Children's Research Hospital.

## Abbreviations

AJCC: American Joint Committee on Cancer
AST: Atypical spitzoid tumor
CLND: Complete lymph node dissection
CM: Conventional melanoma
DFS: Disease-free survival
HR: Hazard ratio
IQR: Interquartile range
M: Median
MSLT: Multicenter Selective Lymphadenectomy Trial
MSS: Melanoma-specific survival
OS: Overall survival
SLN: Sentinel lymph node
SLNB: Sentinel lymph node biopsy
SM: Spitz melanoma
